# A comes before B, like 1 comes before 2. Is the parietal cortex sensitive to ordinal relationships in both numbers and letters? An fMRI‐adaptation study

**DOI:** 10.1002/hbm.24897

**Published:** 2019-12-18

**Authors:** Celia Goffin, Stephan E. Vogel, Michael Slipenkyj, Daniel Ansari

**Affiliations:** ^1^ Numerical Cognition Laboratory, Department of Psychology and Brain and Mind Institute The University of Western Ontario London Ontario Canada; ^2^ Educational Neuroscience Institute of Psychology, University of Graz Graz Austria

**Keywords:** fMRI adaptation, intraparietal sulcus (IPS), number representation, ordinality, symbolic number

## Abstract

How are number symbols (e.g., Arabic digits) represented in the brain? Functional resonance imaging adaptation (fMRI‐A) research has indicated that the intraparietal sulcus (IPS) exhibits a decrease in activation with the repeated presentation of the same number, that is followed by a rebound effect with the presentation of a new number. This rebound effect is modulated by the numerical ratio or difference between presented numbers. It has been suggested that this ratio‐dependent rebound effect is reflective of a link between the symbolic numerical representation system and an approximate magnitude system. Experiment 1 used fMRI‐A to investigate an alternative hypothesis: that the rebound effect observed in the IPS is related to the ordinal relationships between symbols (e.g., 3 comes before 4; C after B). In Experiment 1, adult participants exhibited the predicted distance‐dependent parametric rebound effect bilaterally in the IPS for number symbols during a number adaptation task, however, the same effect was not found anywhere in the brain in response to letters. When numbers were contrasted with letters (numbers > letters), the left intraparietal lobule remained significant. Experiment 2 demonstrated that letter stimuli used in Experiment 1 generated a behavioral distance effect during an active ordinality task, despite the lack of a neural distance effect using fMRI‐A. The current study does not support the hypothesis that general ordinal mechanisms underpin the neural parametric recovery effect in the IPS in response to number symbols. Additional research is needed to further our understanding of mechanisms underlying symbolic numerical representation in the brain.

## 1 INTRODUCTION

Number symbols (e.g., Arabic numerals) are a relatively recent human invention, therefore, it is unlikely that evolution has adapted the human brain to process and represent numbers symbolically (Núñez, 2017). This prompts an important question: how does the brain come to represent numerical symbols?

To date, the precise mechanisms that enable the human brain, over the course of learning and development, to represent and manipulate numerical symbols remain poorly understood (Coolidge & Overmann, 2012). In the present functional neuroimaging study and behavioral study, we investigate whether numerical symbols and letters are represented in similar or different ways.

### 1.1 Involvement of the parietal lobe in number representation

Adult fMRI research has repeatedly shown that the activity in the parietal cortex is correlated with tasks that involve the processing of numerical symbols (e.g., number comparison). In particular, the intraparietal sulcus (IPS) has been highlighted as a key region for symbolic number representation (e.g., Dehaene, Piazza, Pinel, & Cohen, 2003; Holloway, Battista, Vogel, & Ansari, 2013; Notebaert, Nelis, & Reynvoet, 2010; see Ansari, 2008 for a review). Additionally, studies of patients with parietal lesions as well as studies involving transmagnetic stimulation of the parietal area find numerical skills are negatively impacted when the activity in parietal neural regions is interfered with (Cohen Kadosh et al., 2007; Dehaene & Cohen, 1997). In a recent meta‐analysis of fMRI studies, Sokolowski, Fias, Mousa, and Ansari (2016) found that the left superior parietal lobule (SPL) is consistently activated for symbolic (i.e., Arabic digit) numerical processing. To date, the research has converged upon areas in the parietal lobe such as the IPS and SPL as key neural regions for the processing of numerical stimuli.

A limitation of many fMRI studies is that the tasks employed to elicit neuronal activation in response to numerical symbols require that participants compare two numbers (e.g., Holloway, Price, & Ansari, 2010) or perform calculations (e.g., Rivera, Reiss, Eckert, & Menon, 2005). Such active tasks are potentially problematic because it becomes challenging to separate activation related to response selection from that attributable to the processing of numerical symbols. Put differently, rather than attributing parietal activation to numerical representation, it could be argued that the activation observed in these studies is the result of participants being required to select between two or more response options. It is well established that the parietal cortex plays a critical role in motor control and response selection. In view of this, it is perhaps not surprising that Göbel, Johansen‐Berg, Behrens, and Rushworth (2004) found that neural activity during number comparison was difficult to distinguish from control tasks that did not involve processing of numerical symbols, but did require response selection. In other words, the parietal regions often associated with number representation are recruited for response selection processes that do not involve symbolic number processing. Such findings cast legitimate doubt on the notion that the parietal cortex is critical for the representation and processing of numerical symbols. One method that can be used to mitigate such a confound and to investigate the neural correlates of symbolic number in the absence of response selection is to use a passive task design that requires no overt decisional processes.

### 1.2 Functional magnetic resonance adaptation and symbolic numerical representation

The central assumption behind functional magnetic resonance adaptation (fMR‐A) designs is that the repeated presentation of a certain stimulus attribute (e.g., color) will result in the reduction of activation in the neural regions that are critical for processing a given attribute/stimulus characteristic (Grill‐Spector, Henson, & Martin, 2006). A rebound effect can then be observed when another stimulus that differs from the adaptation‐phase stimulus in the attribute of interest—a so‐called “deviant” stimulus—is presented. Upon presentation of the deviant stimulus, the previously reduced activation in the adapted brain region rebounds (i.e., increases).

Using an fMR‐A event‐related design, Notebaert et al. (2010) examined brain activation in response to symbolic number presentation. Participants' brain responses were adapted to either the Arabic digit “6” (small number condition) or the digit “32” (large number condition). Numbers that deviated from the adaptation number were presented randomly throughout the run after the adaptation periods. The left IPS showed a significant ratio‐dependent neural rebound effect for both the small and large number conditions. More specifically, greater activation in the left IPS was revealed for deviants whose ratio with the adapted number was relatively small compared to deviants whose ratio with the adapted number was comparatively large (Notebaert et al., 2010). This ratio‐dependent rebound effect has been replicated by multiple studies (e.g., Holloway et al., 2013; Vogel, Goffin, & Ansari, 2015; Vogel, Goffin, et al., 2017).

fMR‐A research using numerical stimuli has for the most part converged on the finding that the IPS shows a signal recovery effect that is dependent on numerical ratio (Holloway et al., 2013; Notebaert et al., 2010; Piazza, Pinel, Le Bihan, & Dehaene, 2007; Vogel et al., 2015; Vogel, Goffin, et al., 2017). This ratio‐dependent neural rebound effect has been suggested to result from the mapping of the symbolic numerical system onto a noisy, analog system of magnitude representation, called the approximate number system (ANS; Dehaene, 1997). In this ANS account of number representation, number magnitudes are represented on a mental number line in an analog fashion, and symbolic numbers are mapped onto this noisy magnitude system (Dehaene, 1997). Each numerical quantity on this number line is hypothesized to be associated with a distribution of representational uncertainty (e.g., the representation of four also includes that of three and two) around the precise location of the number quantity, resulting in an analog representation of numerical magnitude (Dehaene, 1997). When people are asked to compare two numbers, this analog system of representing number results in a characteristic behavioral signature: the numerical distance effect (NDE; Moyer & Landauer, 1967). The NDE is measured as an increase in reaction time and decrease in accuracy when presented numerical stimuli are numerically closer together, as compared to farther apart. It has been hypothesized that numbers that are numerically closer have more overlap in their distributions (share more of their representational uncertainty) on the mental number line. Increased overlap between these distributions results in the increased reaction time and decreased accuracy observed in the behavioral NDE (Moyer & Landauer, 1967). In a similar vein, overlap in these representations has been proposed to explain the ratio‐dependent rebound effect observed in symbolic number adaptation studies.

However, this theory that symbolic numbers are directly mapped onto the ANS has been challenged within the numerical cognition field (e.g., Lyons, Ansari, & Beilock, 2012). For example, research has called into question the presence of a strong link between symbolic and nonsymbolic numerical systems. Lyons et al. (2012) found a processing “cost” when participants were asked to complete a task involving both symbolic and nonsymbolic stimuli compared to conditions with a single format, suggesting that these formats are not interchangeable without extra processing. Moreover, Lyons, Nuerk, and Ansari (2015) found that measures of acuity for symbolic and nonsymbolic numerical representation were not significantly associated with one another in a sample of elementary school aged children. These findings suggest that number symbols are not necessarily inextricably tied to nonsymbolic quantities, questioning the notion of a direct link from nonsymbolic to symbolic numerical representation. Furthermore, symbolic and nonsymbolic systems may show divergent patterns of representation at the neural level. While nonsymbolic numerical representation can be modeled using a tuning curve function, symbolic numerical representation does not follow this pattern, and instead fits a more precise, non‐analog model (Lyons, Ansari, & Beilock, 2015). A lack of a direct link between nonsymbolic and symbolic behavioral measures and qualitatively different representations at the neural level challenge the ANS theory of symbolic number representation.

*What factors, other than overlap in the representations of analog numerical magnitudes, could explain the ratio‐dependent rebound effects frequently observed for symbolic number?* It could be plausibly hypothesized that instead of being involved in the representation of numerical magnitude, the IPS is engaged by the ordinal associations between numerical stimuli. Numbers can be arranged ordinally; early on children learn that two follows one and three follows two (Butterworth, 2005). Thus, is it possible that the ordinal associations between number stimuli create a recovery effect that mimics what we would see with an analog number representation system? But how can this be examined? In the aforementioned fMR‐A studies it is impossible to distinguish whether adaptation effects are driven by ordinal or ratio‐dependent representations, since the existing data is equally plausible under both accounts (e.g., 2 and 3 have both a larger ratio and have greater ordinal proximity than 2 and 6).

Critically, the use of letters as stimuli provides the opportunity to test whether general ordinal associations underpin the representation of symbolic number in the IPS. Letters can be ordered (i.e., the alphabet) and, as is the case for numbers, children learn this ordinal sequence (e.g., they practice that B follows A and C follows B; Justice, Pence, Bowles, & Wiggins, 2006). As adults, we use an alphabet ordering system for various tasks, such as filing and organizing references. Although letters have ordinal associations, unlike numbers they do not have magnitude associations. The presence of an order system and the absence of a magnitude system make letters ideal stimuli in order to disambiguate between the aforementioned ratio‐dependent and ordinal associations accounts of adaptation of symbolic number in the IPS. More specifically, if there are similarities in the rebound effects for letters and numbers in the IPS, then an ordinal account is more likely. If, however, only numbers exhibit such an effect, then great confidence can be associated with the ratio‐dependent explanation of the rebound from adaptation of the IPS signal to symbolic number.

In view of the above, the aim of the current study was to explore the mechanisms of the distance/ratio‐dependent recovery effect observed in numerical fMR‐A research. Presently, it is unknown whether the parietal recovery effect is specifically modulated by changes in numerical magnitude. Put differently, it is unclear whether the recovery effect observed in the IPS can be unambiguously attributed to the direct mapping of symbolic numbers onto an analog system of magnitude representation, or whether it may be reflective of some other numerical attribute, such as ordinality. The ordinal associations between numbers could generate an effect that is indistinguishable from that which would be generated by overlapping representations of numerical magnitude, thereby resulting in the mistaken attribution of the neural parametric effect to an ANS system of number representation.

With this gap in the literature in mind, the current study will address the following question: Will the IPS show a recovery effect if presented with nonnumerical, ordered stimuli with no magnitude associations? To address this question, we presented adults with symbolic stimuli that have strong ordinal associations: digits and letters. Letters have been shown in previous research to have strong ordinal associations (Jou & Aldridge, 1999), but unlike symbolic numbers, letters do not have a magnitude associated with them.

**Figure 1:**
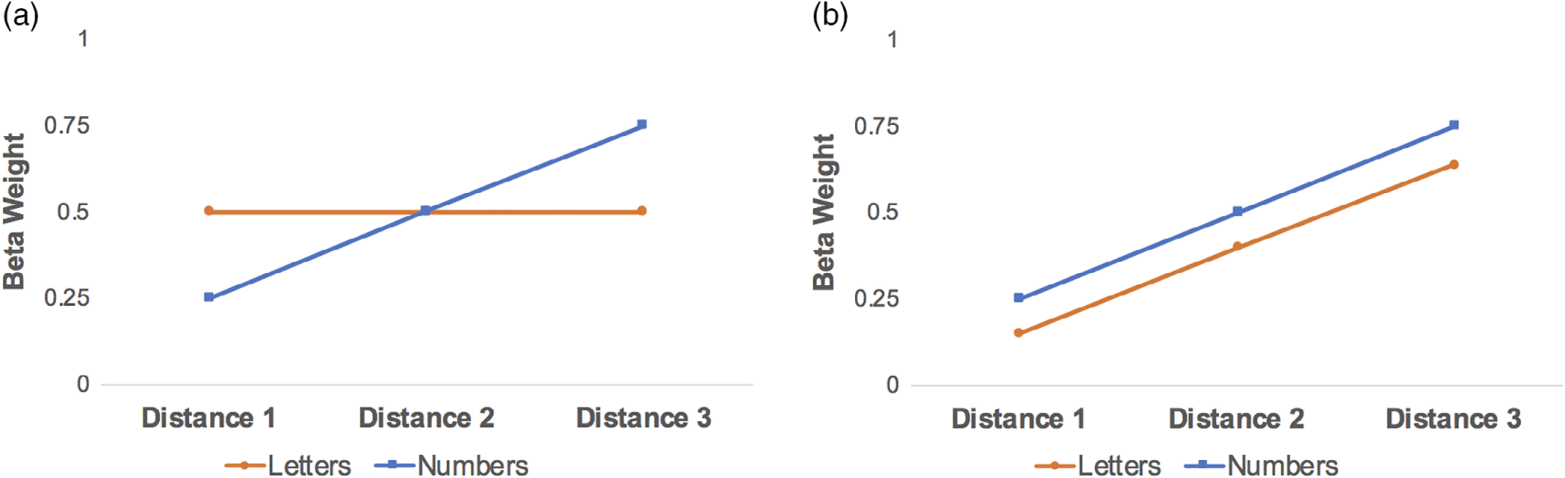
Predictions for parietal activation during the adaptation task for the number (blue) and letter (orange) conditions. Distance represents numerical distance between the adapted value and deviant. (a)Only numbers demonstrate a distance‐dependent rebound effect. This would not support the hypothesis of ordinal mechanisms as underlying the parametric effect, and would suggest this effect is more number‐specific. (b) Both numbers and letters result in a parametric modulation of brain activity. This would suggest that ordinal relationships between symbols could account for the parametric effect

If direct mapping from symbolic digits to nonsymbolic magnitudes can explain the ratio/distance modulated recovery in signal observed in the IPS, symbolic stimuli with no inherent magnitude association should not elicit a parametric effect (Figure 1).

There already exists some data to suggest that there may be similarities in the way in which letters and number are processed in the brain. Specifically, Attout, Fias, Salmon, and Majerus (2014) and Fias, Lammertyn, Caessens, and Orban (2007) found activation in the horizontal section of the IPS in response to both letter and number stimuli, which suggests that the IPS activation observed for numerical stimuli could be at least partially reflective of general ordinal relationships among symbols.

In Experiment 1, we build on the existing evidence and probe whether letters and numbers lead to similar patterns of rebound from adaptation in the IPS. Using letters allows us to disentangle two different mechanisms that could result in similar patterns of activation; representational overlap as predicted by the ANS, and symbol–symbol ordinal associations. Moreover, using a passive design allows us to mitigate the response selection confound that was present in previous studies.

## 2 EXPERIMENT 1

### 2.1 Materials and methods

#### 2.1.1 Participants

Participants were recruited from the University of Western Ontario campus in London, Canada. Twenty‐seven healthy, right‐handed adults with normal or corrected to normal vision participated in this study. In order to be included for analysis, participants had to pass the motion and accuracy criteria for at least one of the two functional adaptation runs. Motion could not exceed 3 mm of drift across the entire run or greater than 1.5 mm jump between successive volumes (Vogel et al., 2015). Runs that did not meet these motion criteria were not included in analysis. Accuracy on the adaptation task catch trials had to be at least 5/7 catch trials.

Three participants were not included in the analysis for the following reasons: one participant experienced claustrophobia and pressed the emergency call button, ending the scanning session before completion, and two participants did not fulfill the accuracy criteria for the adaptation runs, therefore we cannot assume that they were awake for the duration of the run. This left 24 participants ages 19.17–28.08 years (*M*_age_ = 22.78 years; 14 males) for analysis. Informed consent was obtained, participants were compensated monetarily for their time, and were sent a picture of their brain.

#### 2.1.2 Adaptation task

The design of the adaptation task was based on Vogel, Goffin, et al. (2017). The task stimuli consisted of black (R‐G‐B values 0, 0, 0) English letters and Arabic numerals displayed on a gray background (R‐G‐B values 192, 192, 192). The catch trials were presented in red (R‐G‐B value 255, 0, 0). The numbers used were: 2, 3, 4, 5, 6, 7, and 8. The letters corresponding to these numbers were used: B, C, D, E, F, G, and H. In order to minimize adaptation to the visual characteristics of the symbols, two font sizes (sizes 40 pt and 50 pt) and four font types (Times New Roman, Courier New, Calibri, and Arial) were used. Additionally, the location of the symbol varied randomly across six locations, all 2° from the display center (*x*,*y* position from the center = 435, 300; 365, 300; 375, 325; 425,325; 375,275; 425,275). The Eprime 2.0 software was used to project the stimuli onto a screen in the MRI.

**Figure 2:**
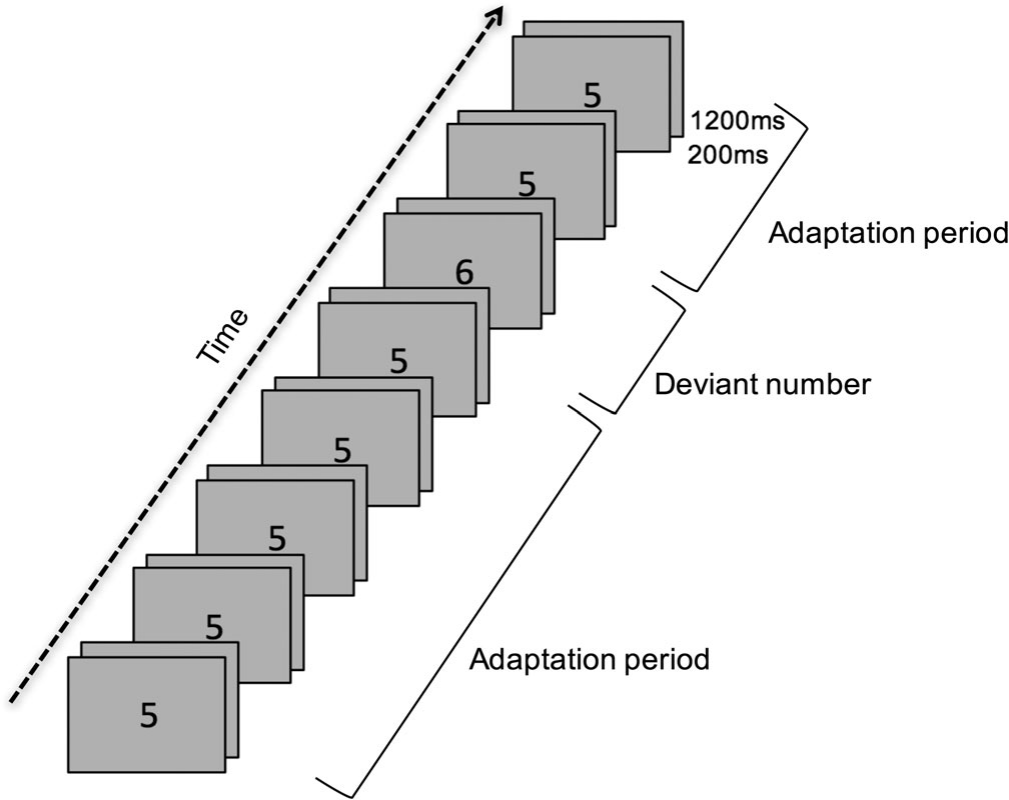
Example of the number condition in the adaptation task. The adaptation period (repeated presentation of 5) is sometimes followed by a deviant number (in this Case 6)

**Figure 3:**
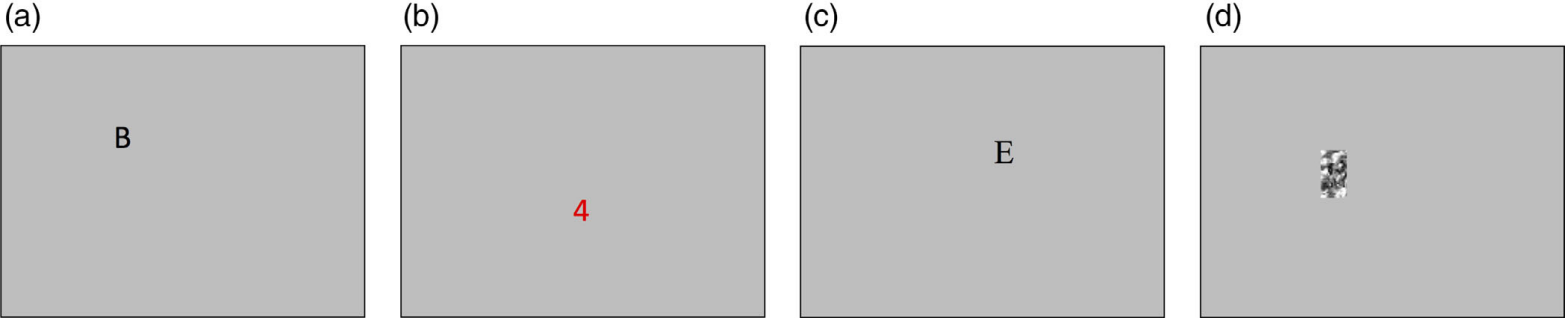
Trial types that followed the adaptation periods. (a) Deviant trial, (b) catch trial, (c) null trial, and (d) scrambled trial

An event‐related design was used. Each symbol appeared on the screen for 200 ms and was followed by a blank screen for 1,200 ms (see Figure 2). Half of each run of the adaptation task was made up of only numbers, and the other half‐only letters. In other words, both the number and letter conditions were presented within each run, separated by a short break (14,000 ms) The order of presentation of the number and letter conditions was counterbalanced across participants. For the number condition the digit 5 was used to habituate brain response, and the corresponding letter E was used for the letter condition. In the adaptation period, the number 5 for the number condition, or the letter E for the letter condition, was repeated between five and nine times, with an average of seven repetitions across the run. The adaptation period was followed by the pseudorandom presentation of one of 48 deviant trials (8 for each numerical/letter deviant), one of 7 catch trials, one of 8 null trials or one of 7 scrambled trials per condition. A pseudorandom order was used in order to ensure that catch trials would appear throughout the duration of the run. Deviant trials differed from the habituation value 5 or E by a distance of 1, 2, or 3 (see Table 1). Catch trials consisted of each of the stimuli used presented in red font and were included to help ensure participants were attending to the stimuli on the screen. Participants were asked to press a button as soon as they saw a red symbol. Null trials consisted of another presentation of the habituation value (i.e., 5 or E). As the null trials were indistinguishable from the adaptation period, these trials were modeled in the baseline for the neural rebound effect. The baseline was used in all contrasts in the whole‐brain analyses to identify regions that demonstrated activation above baseline (the specific contrasts are described in Section 2.1.6). The scrambled stimuli consisted of a Fourier‐transformed version of each of the number and letter stimuli used. These nonsense stimuli were included so as to further control for regions that may show a rebound effect simply for change in visual features. To our knowledge, this is the first number adaptation study to use nonsense symbols as a control for lower‐level perceptual changes. As these scrambled stimuli were not recognizable as a number or a letter, they did not have a semantic meaning. See Figure 3 for an example of each of the stimuli types.

**Table 1:** Stimuli used in the number and letter conditions in the adaptation task

Distance	Numbers		Letters	
0	5		E	
1	4	6	D	F
2	3	7	C	G
3	2	8	B	H

*Note*: Stimuli are sorted by distance from the adaptation symbol (i.e., 5/E).

#### 2.1.3 Procedure

Participants were screened for MRI safety and the task instructions were explained. They were given earplugs to reduce the noise of the scanner and foam cushions were used around the head to reduce head movement. Participants viewed the tasks through a mirror system attached to the head coil of the scanner. For the adaptation task, participants were told that they would see numbers and letters appear on the screen, and to keep their eyes on the screen for the duration of the task. They were shown the button response and told to press the button with their right index finger whenever they saw a red symbol. Participants also completed an arithmetic verification task and a phonology task; however, for the purposes of this article these tasks are not included in the analysis. Participants completed two runs of the adaptation task, and one run each of the arithmetic and phonology tasks. The order of the tasks was counterbalanced across participants, however to reduce fatigue effects the two adaptation runs never directly followed one another. An anatomical scan was collected last. The participants were in the scanner for approximately 1.5 hr. After the scanning session, participants completed a Math Fluency task from the Woodcock Johnson III Tests of Achievement as well as a phonology verification task; however, these tasks were not analyzed for the purposes of the current article. The entire testing session took no more than 2 hr.

#### 2.1.4 fMRI data acquisition

Functional and anatomical data were collected with a 3T Siemens Magnetom Prisma scanner at the Robarts Research Institute in London, Canada using a Siemens 32‐channel head coil. fMR‐A data were collected with a BOLD‐sensitive T2*‐weighted echo planar (EPI SE) sequence. Thirty‐five slices per volume were acquired covering the whole brain using an ascending‐interleaved method (3 mm thickness, 70 × 70 matrix; field of view = 210 × 210 mm; TR = 2,000 ms; echo time = 57 ms; flip angle = 78°). For the adaptation task, 860 volumes per functional run were acquired. Each run was 28 min and 40 s long.

High‐resolution T1‐weighted MRI data were collected at the end of the functional runs in the sagittal plane (voxel size of 1 mm × 1 mm × 1 mm; 192 slices; TR = 2,300 ms).

#### 2.1.5 fMRI analysis

Functional data were preprocessed using the Brainvoyager 20.6 software (Brain innovation, Maastricht, The Netherlands). Functional data were corrected for head motion, low frequency noise, and differences in slice scan‐time acquisition and spatially smoothed with a 6 mm FWHM Gaussian smoothing kernel.

Functional imaging data were aligned with the anatomical data. The anatomical data and functional runs were transformed into MNI‐152 space for analysis at the group level. The hemodynamic response was modeled using a 2‐gamma function. A whole‐brain, random effects general linear model (GLM) was then used. An uncorrected threshold of *p* < .005 was used to find neural regions active for each analysis. Cluster correction was then used to correct for multiple comparisons (Forman et al., 1995; Goebel, Esposito, & Formisano, 2006) at the whole‐brain level. A mask of the whole brain was used to restrict the cluster calculation to voxels inside the brain. A Monte‐Carlo algorithm with 1,000 iterations was used to determine the minimum size of a cluster that would result in a false positive rate of 5% (Goebel et al., 2006). The cluster correction was then carried out at a whole‐brain level and clusters that remained at a threshold of *p* < .05 (cluster‐corrected) were identified as significant.

#### 2.1.6 Data analysis

As a first step, accuracy on the adaptation task catch trials was examined, resulting in any run scoring below 71.4% (5/7 catch trials) being removed from further analysis. This number was chosen to match as closely as possible to the accuracy cutoff used in previous studies (e.g., Vogel et al., 2015: cutoff = 6/8 catch trials, or 75%).

To examine the presence of a neural distance‐dependent rebound effect for letters or numbers, parametric predictors were created for each participant. Using the deviant stimuli, predictors were weighted for Distances 1 (4 and 6; D and F), 2 (3 and 7; C and G) and 3 (2 and 8; B and H) in relation to the adaptation symbol (5/E). The parametric predictors were created for the number condition (i.e., distance effect for number) and the letter condition (i.e., distance effect for letter). The weighted deviant trials were entered as parametric regressors into a GLM (Holloway et al., 2013). The parametric predictors allowed us to identify regions with a distance‐dependent recovery effect. More specifically, this model predicts an increase in signal recovery with an increase in distance from the adaptation symbol. This analysis is similar to analyses used by Holloway et al. (2013), Vogel et al. (2015) and Vogel, Goffin, et al. (2017). A predictor for catch trials was also created. This predictor was entered into the GLM as a predictor of no interest to account for additional variance in the model (Vogel et al., 2015). The baseline was modeled on the adaptation and null stimuli. The recovery effect was evaluated by looking at the signal change from baseline with the presentation of a deviant.

Using the parametric predictors described above, whole‐brain multisubject GLMs were run. We looked for regions that exhibited distance‐dependent recovery of activation for the letter and number deviants. To identify these regions, the following contrasts were run: parametric effect of deviant_Letter_ > baseline and parametric effect of deviant_Number_ > baseline. This analysis will identify regions that show a distance‐dependent recovery in activation (parametric distance effect). Based on previous number adaptation literature, we expect to find a parametric recovery effect in the left IPS for both the letter and number stimuli. Next, we examined any differences between the letter and number conditions: parametric effect of parametric effect of deviant_Number_ > parametric effect of deviant_Letter_. Within FSLview, the MNI standard map (avg152T1_brain.nii.gz) was loaded and peak coordinates and center of gravity coordinates were entered in MNI space. Brain regions were then identified using the Jülich Histological Atlas (Eickhoff et al., 2005) and Harvard‐Oxford Cortical Structural Atlas (Harvard‐Oxford Cortical Structural Atlas, RRID:SCR_001476) within the FSLview software (Smith et al., 2004).

Main effects for the letter and number deviants were modeled in order to identify brain regions that show any recovery effect due to a change in stimulus. For this purpose, the following contrasts were used: main effect of deviant_Number_ > baseline, main effect of deviant_Letter_ > baseline. For these contrasts, we expected to see IPS activation as well as visual and frontal regions involved in attention and change detection.

A main effect predictor was also calculated for the scrambled stimuli. We used the scrambled symbol events to investigate whether regions identified in the deviant number and letter main effect were responding to the meaning of the symbols, or rather a change in visual properties. In other words, if the main effect for the meaningful symbols (i.e., letters and numbers) identifies regions that show activation over and above that shown for the scrambled stimuli that would suggest that regions demonstrating a main effect may be involved in representation of the symbols. However, if there are no regions that demonstrate greater activation for the main effect versus the scrambled main effect, this would suggest the symbol main effect is reflective of some sort of change detection mechanism. Therefore, to look for regions that demonstrate a recovery effect specific to meaningful symbols (rather than simply deviants in visual properties) the following contrasts were calculated: main effect of deviant_Number_ > main effect of number scrambled symbols_Number_, main effect of deviant_Letter_ > main effect of letter scrambled symbols_Letter_. Activation in the IPS and frontal regions was predicted for both of these contrasts.

### 2.2 Results

#### 2.2.1 Behavioral results

To be included in the analyses, participants had to catch at least 5 of 7 catch trials in each condition of each run. Each participant completed two runs of the adaptation task. Of the 24 participants that had at least one run of the adaptation task that fulfilled the motion and accuracy criteria, five runs were not included because they exceeded the motion cutoff, and four runs were not included because they did not fulfill the accuracy cutoff. This left 39 runs in total for the analysis. Accuracy on these runs had a mean of 0.97, *SD* = 0.06.

#### 2.2.2 Imaging results

To identify regions of the brain that respond to any deviation in the number or letter stimuli, the main effect of the deviants for each condition was contrasted against the baseline activation. This analysis models all deviant symbols as the same; in other words, the deviants are not modeled according to their distance from the adapted symbol. At the whole‐brain level, two clusters in the visual cortex were significant after cluster correction for the contrast main effect for numbers > baseline (Table 2). For the contrast main effect for letters > baseline, five clusters reached significance (Table 3).

**Table 2:** Location of significant clusters identified at the whole‐brain level for the main effect of number deviants

Region—Center of gravity	Hemisphere	Mean *x*	Mean *y*	Mean *z*	*SD x*	*SD y*	*SD z*	Cluster size	Region—Peak voxel	*x*	*y*	*z*	*t*	*p*
Visual cortex V3V	Right	31.05	−87.78	−2.76	6.97	3.91	5.44	848	Visual cortex V4	39	−86	−8	4.360484	.000229
Visual cortex V3V	Left	−23.91	−90.67	−6.7	6.09	4.91	5.31	2,621	Visual cortex V2 BA18	−24	−97	−8	4.070763	.000472

*Note*: Coordinates are in MNI space. Cluster size is given in number of voxels. Regions were identified using the Jülich Histological Atlas (Eickhoff et al., 2005).

**Table 3:** Location of significant clusters identified at the whole‐brain level for the main effect of letter deviants

Region—Center of gravity	Hemisphere	Mean x	Mean y	Mean z	*SD* x	*SD* y	*SD* z	Cluster size	Region—Peak voxel	*x*	*y*	*z*	*t*	*p*
Visual cortex V1 BA17	Right	0.05	−74.71	0.51	30.31	15.15	15.87	80,661	Visual cortex V4	45	−67	−14	8.716191	<.000001
Anterior IPS hIP1	Left	−25.39	−65.7	40.91	3.42	7.3	9.12	6,263	Superior parietal lobule 7A	−27	−70	34	5.443437	.000016
Frontal orbital cortex*	Left	−30.01	29.01	1.17	3.48	4.66	3.47	1,200	Insular cortex*	−30	29	4	4.254945	.000298
Broca's area BA44	Left	−41.19	22.94	17.52	4.65	2.55	5.71	1,090	Broca's area BA45	−39	26	13	4.353102	.000234
Broca's area BA44	Left	−45.01	4.83	33.86	6.29	5.12	2.85	3,058	Corticospinal tract	−39	−1	34	5.528336	.000013

*Note*: Coordinates are in MNI space. Cluster size is given in number of voxels. Regions were identified using the Jülich Histological Atlas (Eickhoff et al., 2005) unless no region was found in this atlas for the specified coordinates. These regions (marked with *) were identified using the Harvard‐Oxford Cortical Structural Atlas (Harvard‐Oxford Cortical Structural Atlas, RRID:SCR_001476).

**Figure 4:**
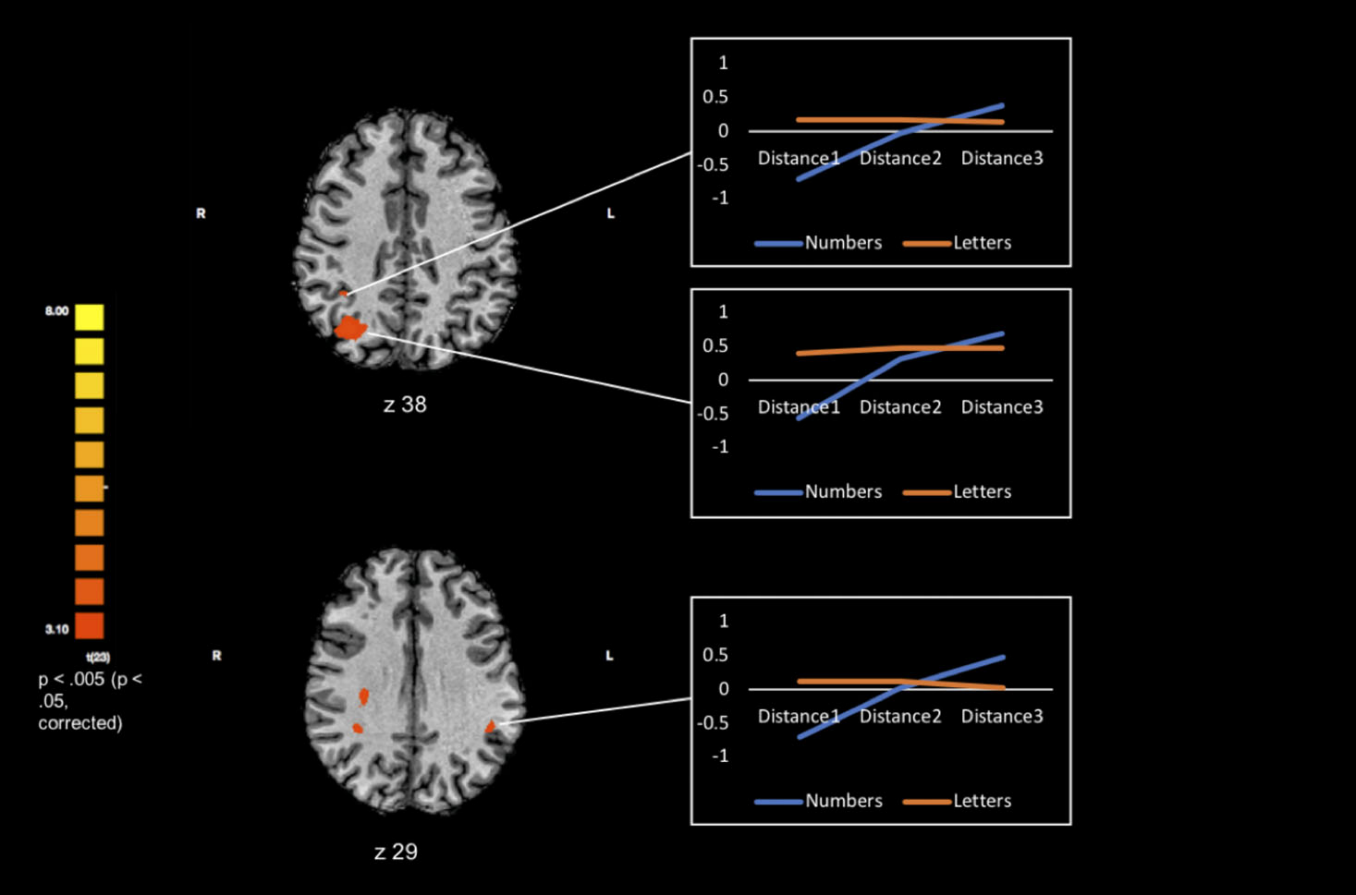
Right anterior IPS clusters and left anterior IPS are activated for the number parametric effect. Coordinates are in MNI space. The line graphs represent the distance‐dependent modulation for numbers (blue) and letters (orange) in the right anterior IPS clusters (top) and left anterior IPS. These points were derived by extracting the beta weights from the parietal regions that exhibited a significant parametric effect for numbers. Numbers demonstrate the predicted distance‐dependent parametric increase of rebound of activation, whereas letters do not demonstrate this pattern

Next, the whole brain was examined for a distance‐dependent parametric recovery effect for each of the number and letter conditions. For the contrast parametric regressor for numbers > baseline, four significant clusters were identified (see Table 4). Most notably, clusters in the right anterior IPS and left anterior IPS were found to show the expected distance‐dependent activation pattern (see Figure 4). No significant regions were found to show a parametric effect for the parametric regressor for letters > baseline contrast. Moreover, even at an increased threshold of .01 uncorrected, no regions demonstrated a parametric effect for letters.

**Table 4:** Location of significant clusters identified at the whole‐brain level for the parametric effect of number deviants

Region—Center of gravity	Hemisphere	Mean x	Mean y	Mean z	*SD* x	*SD* y	*SD* z	Cluster size	Region—Peak voxel	*x*	*y*	*z*	*t*	*p*
Anterior IPS HIP1	Right	33.28	−65.29	37.8	4.07	3.1	2.63	1,313	Anterior IPS HIP1	33	−67	37	4.261469	.000293
Anterior IPS HIP2	Right	33.13	−37.25	32.86	3.15	7.6	2.54	1,365	Anterior IPS HIP3	33	−40	34	4.063882	.00048
Premotor cortex BA6	Right	28.39	1.39	48.96	3.05	4.58	3.52	1,262	Premotor cortex BA6	33	−4	49	4.385268	.000216
Optic radiation	Left	−38.71	−45.68	20.15	5.29	2.06	5.65	945	Anterior IPS HIP1	−39	−46	22	4.472119	.000174

*Note*: Coordinates are in MNI space. Cluster size is given in number of voxels. Regions were identified using the Jülich Histological Atlas (Eickhoff et al., 2005).

**Figure 5:**
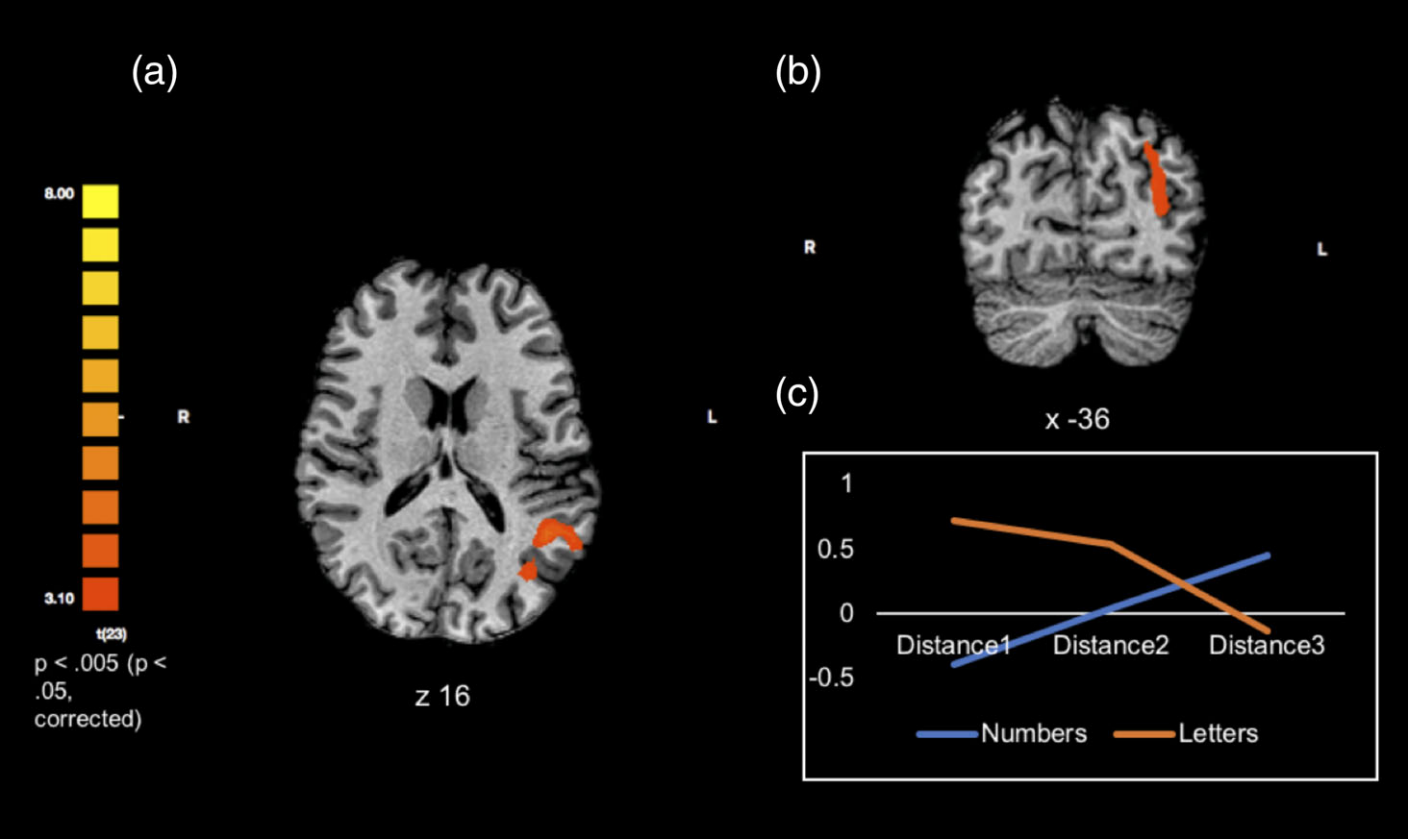
Significant parietal cluster for the contrast number deviant parametric effect > letter deviant parametric effect. (a) Transverse view of statistically significant parietal cluster. (b) Coronal view of statistically significant parietal cluster. (c) The line graph represents the distance‐dependent modulation for numbers (blue) and letters (orange) in the left inferior parietal lobule. These points were derived by extracting the beta weights from the parietal regions that exhibited a significant parametric effect for numbers. Numbers demonstrate the predicted distance‐dependent parametric increase of rebound of activation, whereas letters do not demonstrate this pattern

To further investigate the specificity of the parametric effect for numbers, the following contrast was carried out at the whole‐brain level: parametric effect for numbers > parametric effect for letters. Two significant clusters were found including the left inferior parietal lobule (see Table 5 and Figure 5).

**Table 5:** Location of significant clusters identified at the whole‐brain level for the parametric effect of number deviants > parametric effect of letter deviants

Region—Center of gravity	Hemisphere	Mean x	Mean y	Mean z	*SD* x	*SD* y	*SD* z	Cluster size	Region—Peak voxel	*x*	*y*	*z*	*t*	*p*
Inferior parietal lobule PGp	Left	−35.49	−70.91	26.36	2.9	2.24	8.94	1,210	Optic radiation	−33	−70	25	4.031529	0.00052
Middle temporal gyrus, temporoocipital part*	Left	−48.11	−50.64	13.88	5.17	4.2	2.37	1,491	Angular gyrus*	−45	−52	16	4.440388	0.000188

*Note*: Coordinates are in MNI space. Cluster size is given in number of voxels. Regions were identified using the Jülich Histological Atlas (Eickhoff et al., 2005) unless no region was found in this atlas for the specified coordinates. These regions (marked with *) were identified using the Harvard‐Oxford Cortical Structural Atlas (Harvard*‐*Oxford Cortical Structural Atlas, RRID:SCR_001476).

Contrasts with the scrambled symbolic stimuli were also examined at the whole‐brain level in order to better understand the main effect findings. In particular, because the scrambled stimuli have no meaning, if the number and letter main effects are contrasted with the scrambled stimuli we can examine whether the main effects are related to a change detection mechanism, as opposed to processes related to symbol processing. More specifically, if the main effect for numbers and letters reflect any stimulus specific processing, then these main effects should show greater activation for either letters or number relative to the scrambled conditions. If, however, the main effects are mostly reflective of general processes such as change detection and a change in attentional state, then there should be no regions that show a greater main effect for letters or numbers compared to the scrambled symbols. Indeed, this is what we found. For the contrast of main effect of numbers > main effect of scrambled numbers, there were five regions that were greater for the scrambled stimuli (i.e., showed greater activation for scrambled numbers compared to numbers; i.e., negative *t*‐values for the contrast main effect of numbers > main effect of scrambled numbers; Table 6). Similarly for the letter main effect > main effect of scrambled letters, five clusters demonstrated negative activation (Table 7). These findings are convergent with a change detection explanation of the main effects observed, rather than processing of symbol‐specific information. If the main effects were specifically associated with symbolic processing, we might expect to see activation for the numbers and letters that is greater than the activation for the scrambled stimuli. Instead, there is evidence for more robust activation in response to the scrambled symbols that carry no semantic meaning but greater novelty. Whatever may explain the greater activation for scrambled symbols, the evidence does not point to the main effects being reflective of stimulus‐specific activation patterns.

**Table 6:** Location of peak voxels for significant clusters identified at the whole‐brain level for the main effect of number deviants > main effect of scrambled numbers

Region—Center of gravity	Hemisphere	Mean x	Mean y	Mean z	*SD* x	*SD* y	*SD* z	Cluster size	Region—Peak voxel	*x*	*y*	*z*	*t*	*p*
Visual cortex V4	Right	39.57	−63.58	−10.72	6.98	13.9	7.11	15,153	Temporal occipital fusiform cortex*	42	−58	−11	−7.259053	<.000001
Broca's area BA44	Right	41.79	9.21	31.31	3.96	3.7	3.83	2,132	Broca's area BA44	42	8	31	−5.239583	.000026
Interior occipito‐frontal fascicle	Right	31.64	23.91	−0.92	3.4	4.64	3.08	945	Inferior occipito‐frontal fascicle	27	26	1	−3.896417	.000727
Anterior IPS HIP3	Right	34.37	−58.55	44.78	2.16	4.39	2.75	962	Anterior IPS HIP3	33	−61	46	−4.022963	.000531
Occipital fusiform gyrus*	Left	−40.26	−65.22	−11.4	6.49	11.87	5.88	14,527	Lateral occipital cortex, inferior division*	−45	−67	−11	−7.163185	<.000001

*Note*: Coordinates are in MNI space. Cluster size is given in number of voxels. Regions were identified using the Jülich Histological Atlas (Eickhoff et al., 2005) unless no region was found in this atlas for the specified coordinates. These regions (marked with *) were identified using the Harvard‐Oxford Cortical Structural Atlas (Harvard*‐*Oxford Cortical Structural Atlas, RRID:SCR_001476).

**Table 7:** Location of peak voxels for significant clusters identified at the whole‐brain level for the main effect of letter deviants > main effect of scrambled letters

Region—Center of gravity	Hemisphere	Mean *x*	Mean *y*	Mean *z*	*SD x*	*SD y*	*SD z*	Cluster size	Region—Peak voxel	*x*	*y*	*z*	*t*	*p*
Visual cortex V5	Right	41.4	−60.2	−8.33	10	15.04	9.64	17,237	Temporal occipital fusiform cortex*	39	−55	−20	−8.193955	<.000001
Broca's area BA44	Right	43.7	16.9	24.59	6.57	12.48	12.39	14,148	Broca's area BA44	48	11	28	−7.776074	<.000001
Anterior IPS HIP1	Right	31.95	−64.6	39.38	3.21	7.04	8.25	3,077	Superior parietal lobule 7P/anterior IPS HIP1	30	−67	40	−4.511512	.000157
Premotor cortex BA6	Right	7.1	16.75	47.86	2.06	5.57	3.42	992	Premotor cortex BA6	9	23	43	−4.259583	.000295
Visual cortex V4	Left	−37.06	−61.83	−13.74	6.14	12.64	5.02	9,535	Temporal fusiform cortex, posterior division*	−36	−40	−24	−7.38112	<.000001

*Note*: Coordinates are in MNI space. Cluster size is given in number of voxels. Regions were identified using the Jülich Histological Atlas (Eickhoff et al., 2005) unless no region was found in this atlas for the specified coordinates. These regions (marked with *) were identified using the Harvard‐Oxford Cortical Structural Atlas (Harvard*‐*Oxford Cortical Structural Atlas, RRID:SCR_001476).

Contrary to our predictions, letters did not exhibit a distance related parametric effect in any brain region, even at very liberal statistical thresholds (i.e., .01). However, an absence of evidence does not imply evidence for absence. In view of this, in order to further constrain our understanding of the null results obtained for the parametric effect of letters, we quantified the evidence for the null hypothesis (no parametric distance effect for letters) using Bayesian statistics. Specifically, an ROI analysis was conducted using the parietal clusters identified for the number parametric effect > baseline analysis. Beta weights for the letter parametric effect were extracted from the right anterior IPS HIP1 (*M* = 0.04, *SD* = 0.75), right anterior IPS HIP2 (*M* = −0.02, *SD* = 0.80), and left anterior IPS HIP1 (*M* = −0.05, *SD* = 0.66). Using JASP, a Bayesian one‐sample *t* test was then run to determine the strength of the evidence, or Bayes Factor, for the null hypothesis (BF_01_); that is, that there was not a significant parametric effect for letters (JASP Team, 2019). The parametric effect for letters was not found to be significant for the right anterior IPS HIP1, *t*(23) = 0.27, *p* = .792, BF_01_ = 4.51, right anterior IPS HIP2, *t*(23) = −0.12, *p* = .903, BF_01_ = 4.63, or left anterior IPS HIP1, *t*(23) = −0.41, *p* = .688, BF_01_ = 4.32. Overall, the Bayesian *t* tests indicated substantial support in favor of the null hypothesis (Jeffreys, 1961).

## 3 DISCUSSION

Which mechanisms underlie the parametric effect observed in numerical adaptation studies? Experiment 1 used fMR‐A to test whether this effect is driven by an analog system of magnitude representation or whether it can, at least in part, be explained by general processing of ordinal relationships. This was tested by examining the neural adaptation to letters and numbers, which are both ordinal sequences, but numbers, unlike letters, carry information about numerical magnitude as well as numerical order. Bilateral regions in the IPS were shown to be modulated by numerical distance when participants were presented with number symbols. Contrary to the account that posits that the processing of general ordinal associations (e.g., the fact that 1 come before 2 like A comes before B) can account for the adaptation of the IPS to numerical symbols, letters were not found to be associated with a parametric effect anywhere in the brain. Put differently, following adaptation, the ordinal distance between the adapted and deviant letters was not found to modulate brain activation. Finally, when compared to letters, the left inferior parietal lobule was found to be more strongly correlated with the parametric processing of numerical deviants.

Against the background of the findings from Experiment 1, we did not find support for the hypothesis that the parametric effect in the IPS in response to symbolic number can be explained by the processing of ordinal relationships that exist for both letters and numbers. Such an account would have been supported if the parametric response to letters and number was similar. However, presenting participants with letters—symbols that have ordinal associations but no magnitude associations—did not result in a parametric effect. If symbol–symbol ordinal relationships could explain the neural parametric effect observed in the parietal lobe in numerical adaptation studies, presenting participants with letters in an analogous task should have generated a pattern similar to that revealed for number symbols. However, results from Experiment 1 do not provide evidence in support of this hypothesis. Of course, it is also possible that there are differences in the relative degree to which the ordinal associations get activated when participants view a number versus a letter. Perhaps there are different levels to the automaticity with which we access internal representation of such ordinal relationships; with ordinal associations being activated more automatically for numbers, and less automatically for letters. This could also explain the lack of a parametric effect observed for letters.

It is important to highlight that these findings therefore do not refute the ANS theory of symbolic number representation. However, it should be noted that these results also do not provide direct support for the ANS theory either. The current study was not designed to explicitly test the theory of an analog number system as underlying symbolic numerical representation; only to test whether a general representation of order (for both letters and numbers) could account for the data observed. Although ordinality could not explain the parametric effect, it remains to be seen whether a different mechanism can explain the parametric effect for number symbols. For example, perhaps ordinal associations underlie this effect, but the ordinal associations between these symbols must be processed fluently and automatically in order to generate the parametric effect in a passive task (Vogel et al., 2019). Gevers, Reynvoet, and Fias (2003) found that the ordinal position of letter stimuli in an active task influenced task performance when participants completed an ordinal decision task. In a non‐ordinal decision task, the ordinal position of letters also influenced performance, although to a lesser extent, even though ordinality was irrelevant to the task‐at‐hand. Perhaps some level of effortful processing of the letter stimuli, even if this processing is unrelated to ordinality, is necessary to invoke the ordinal representations between letters. Further research that empirically tests alternative mechanisms is necessary to rule out other possible accounts.

In contrast to the present findings, previous research using a letter ordinality task demonstrated bilateral activation in the IPS (Fulbright, Manson, Skudlarski, Lacadie, & Gore, 2003). Specifically, Fulbright et al. (2003) found a network of regions including bilateral IPS to be more activated for letter ordering than identification. While the present results also revealed activation of the left IPS when contrasting the presentation of letter deviants against rest (i.e., the main effect for letters), the interpretation of such an effect is not straightforward. This is because the main effect analysis treats all deviants as the same (i.e., the deviants are not parametrically weighted), thereby making it difficult to distinguish between brain activation due to processing of ordinal position of the letters or something such as change detection. To further demonstrate the lack of specificity of the main effect, when the main effect for letter stimuli was contrasted with the scrambled letter condition, there were no regions that showed greater activation for letters than for the nonsense‐scrambled condition. Because the scrambled condition stimuli were not identifiable as letters, this supports the interpretation that the letter main effect that was observed can likely be attributed to the detection of a change in visual stimulus, as opposed to ordinal processing of the letter stimuli or indeed anything specific to the processing of letters. This converges with findings demonstrating a key role for the IPS in visuo‐spatial attention and suggests that the parietal activation observed in the main effect contrasts likely reflects domain‐general visuo‐spatial attention (e.g., Materna, Dicke, & Thier, 2008; Silk, Bellgrove, Wrafter, Mattingley, & Cunnington, 2010). Examining the brain for regions that show a parametric increase in rebound of activation is therefore a more precise measure of any processing of ordinality rather than the main effect, which most likely reflects activation that is not stimulus specific, such as change detection, or a change in attentional state for example.

## 4 EXPERIMENT 2

### 4.1 Introduction

Experiment 1 tested the hypothesis that the processing of ordinal mechanisms drives the neural parametric effect that has been repeatedly observed in numerical adaptation tasks. Although the parametric effect for numbers was replicated, letters did not exhibit a similar pattern; a finding that does not support such an account. Even though a parametric effect for letters was not obtained at the neural level with a passive task, based on previous research we would still expect letters to generate a behavioral distance effect (Van Opstal, Gevers, De Moor, & Verguts, 2008). In a behavioral study, Van Opstal et al. (2008) used letters to challenge the theory that representational overlap underlies the NDE. When participants were asked to complete a comparison task, an NDE was obtained for both the number and letter condition. The NDE was thus attributed to processes related to response selection, as opposed to a specific numerical process (Van Opstal et al., 2008).

In view of this, the first goal of Experiment 2 was to test whether a distance effect can be obtained with the specific letter stimuli used in Experiment 1. In the absence of such data, it is plausible to posit that the lack of a neural parametric effect may reflect an inability to process the ordinal association between letters. More specifically, if a behavioral distance effect is not obtained with these letter stimuli, perhaps the letters included do not elicit the processing of sequential order. However, if a behavioral distance effect is obtained with the letter stimuli, this would support the notion that there exists a dissociation between the neural parametric effect and the behavioral NDE. To this end, a between‐groups design was used in which participants were randomly assigned to complete an ordinality comparison task in either the number condition or the letter condition. Based on previous research, demonstrating distance effects with letter stimuli, we expected to find distance effects for both the number and letter conditions (Van Opstal et al., 2008).

The second goal of Experiment 2 was to probe whether participants used a numerical magnitude strategy to complete the letter ordinality task. Importantly, if a distance effect is generated with a task using letter stimuli, it could be argued that participants were using a numerical magnitude strategy, in which they assigned a numerical value to each letter in order to complete the letter task (e.g., B = 2). To test this, a letter arithmetic task was used, in which participants were explicitly instructed to assign numerical values to letters to solve a letter arithmetic problem. To test whether completion of the letter condition in the ordinality task involved the use of a numerical magnitude strategy, performance on a letter arithmetic task was compared between two groups: a number and a letter group. In the letter arithmetic task, participants were asked to verify the correctness of arithmetic problems presented with letters (e.g., B + C = E?). If participants are using a numerical assignment strategy to complete the letter behavioral task (e.g., B = 2), one might expect better performance on the letter arithmetic task in the group that practiced the letter ordinality task (i.e., the letter group) when compared to a group that did not practice letter ordinality (i.e., the number group). This is because the letter arithmetic task explicitly asks participants to use a numerical assignment strategy. However, if performance on the letter arithmetic task is not enhanced in the letter group, it is more likely participants are completing the letter behavioral task using the ordinal associations between letters, as opposed to assigning numerical quantities to the letters.

The methods of Experiment 2 were pre‐registered on the Open Science Framework (OSF). Additional preregistered analyses with these data not relevant to the current manuscript are also available on the OSF page (https://osf.io/s6e7u/).

### 4.2 Materials and methods

#### 4.2.1 Participants

Data from two groups of participants were collected for this study: a letter training group and a number training group. Two participants were excluded because of incomplete data collection. This left a total of 184 participants for analysis: 90 in the letter training group (64 females; *M*_age_ = 22.97 years; *SD*_age_ = 3.99) and 94 in the number training group (60 females; *M*_age_ = 22.63 years; *SD*_age_ = 3.34). The sample size was calculated using a Bayesian stopping point described below in the results section (Marsman & Wagenmakers, 2017).

#### 4.2.2 Procedure

Participants completed the following tasks in this order:

1. Four runs of ordinality training with a comparison to standard task (either letters or numbers depending on training group).
2. Letter arithmetic task
3. Number arithmetic task
4. Ordinality task of not‐trained format (either numbers or letters depending on training group).

For the purposes of the current article, the number arithmetic task (Task 3) and ordinality task of not‐trained format (Task 4) were not analyzed, as the focus of the current study was whether or not distance effects could be obtained with the letter stimuli (Task 1 for the letter group), and in turn how each trained condition (letters or numbers) influenced performance on the letter arithmetic task. A fixed order of the tasks was used so that the letter arithmetic task always followed the four runs of training with the ordinality task.

In the ordinality training tasks (Task 1), participants were presented with a number or a letter in the center of the computer screen (5,000 ms or until response, followed by a fixation point, 1,000 ms). They were asked to judge as quickly and as accurately as possible whether the randomly presented number comes before or after 5, or whether the randomly presented letter comes before or after E. Stimuli with distances 1, 2, and 3 from 5/E were used (see Table 1). A total of 192 trials were used per run. In the letter arithmetic task, participants saw an addition (12 problems) or subtraction problem (12 problems) with a solution on the screen (30,000 ms or until response), using the letter stimuli listed in Table 1. Participants indicated as quickly and as accurately as possible, whether the solution was correct or incorrect. Participants were instructed to treat the letters as if they represent their corresponding numerical value (e.g., B = 2).

#### 4.2.3 Results

Analyses were carried out using SPSS software for the frequentist statistics and JASP (JASP Team, 2019) for the Bayesian statistics. First, trials for which reaction time was greater/less‐than three *SD*s from the participant's mean reaction time were removed from analysis, as were all trials with reaction time less than 100 ms (Goffin & Ansari, 2016). This outlier analysis was conducted so as to reduce the inclusion of trials in which participants likely responded without processing the stimuli (unusually low response time), or were not attending to the task (unusually high response time). Next, accuracy for each task was examined (collapsed across groups) and participants who scored below three *SD*s from the mean accuracy on that task were not included in analyses involving that task. This resulted in the following participants being removed: two participants from Run 1 of the ordinality task, three participants from Run 2, four participants from Run 3, four participants from Run 4, and five participants from the letter arithmetic task.

**Figure 6:**
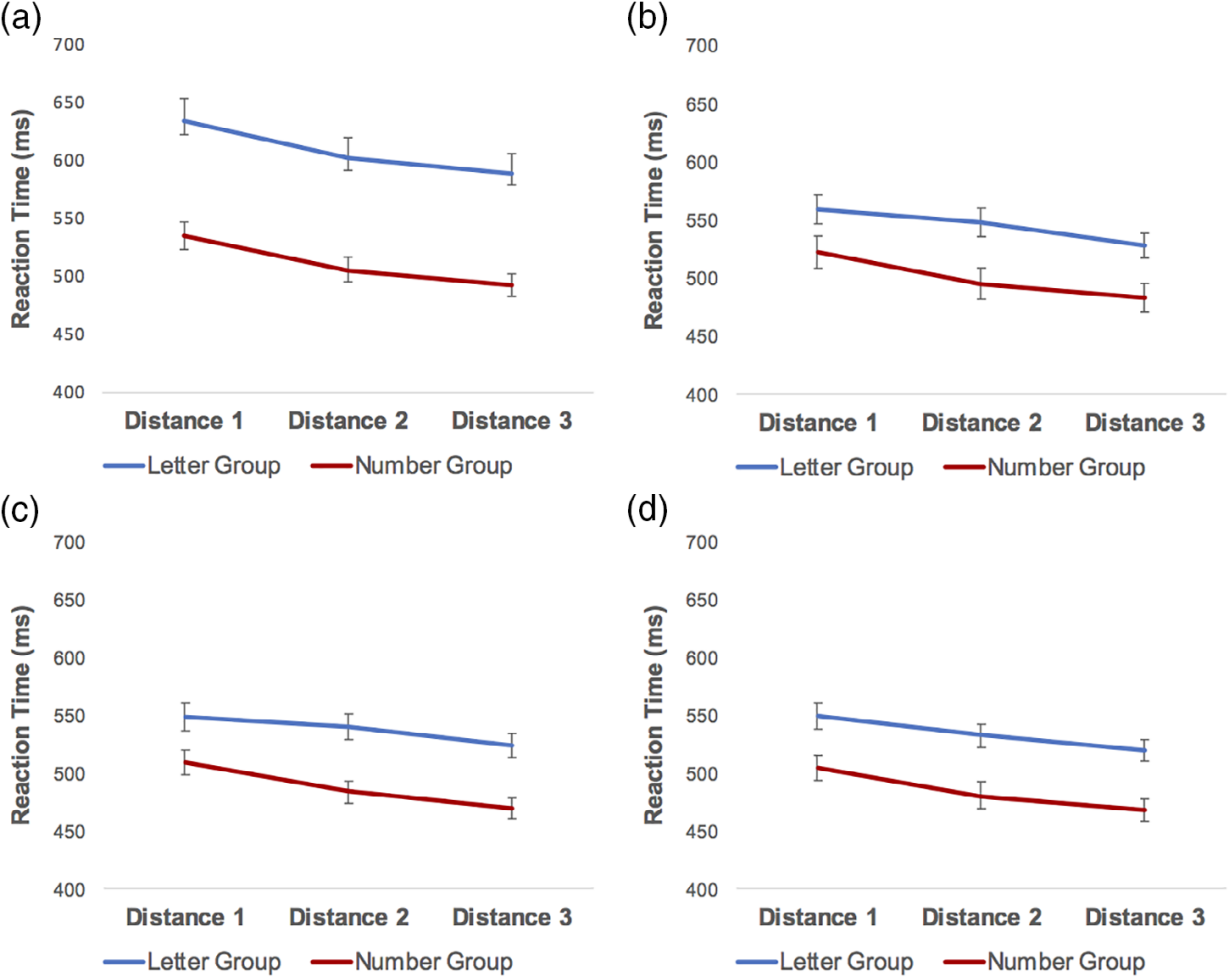
Mean reaction time (ms) for correct trials for distances 1, 2, and 3 on Run 1 (a), Run 2 (b), Run 3 (c), and Run 4 (d) of the ordinality training task for the letter (blue) and number (red) groups. Bars indicate *SEM*. Both groups demonstrated decreased reaction time with increased distance for all four runs

Accuracy was near ceiling for both the letter and number ordinality‐training task (Table 8). Therefore, reaction time data analyses included only correct trials. To examine the effect of distance on the reaction time data, distance effects were calculated using the numerical distance between the presented symbol and the standard symbol (5 or E depending on number or letter condition) for each participant. For this purpose, we used a regression analysis with distance (1, 2, and 3) as a predictor to estimate an individual distance effect for every subject (De Smedt, Verschaffel, & Ghesquière, 2009; Sasanguie, Smedt, Defever, & Reynvoet, 2012; Vanbinst, Ghesquiere, & De Smedt, 2012).The regression slope is an indicator of the size of the distance effect; the larger the regression slope value, the greater the size of the distance effect (Table 9). These standardized regression slopes were then tested against 0 with a one‐sample *t* test to determine whether a significant distance effect was present. Participants in both the number and letter groups demonstrated a negative slope; indicative of decreased reaction time as a function of increasing numerical distance between the presented symbol and the standard in all four runs, in the letter, *t*_Run1_(89) = −11.50, *p* < .001.; *t*_Run2_(89) = −9.44, *p* < .001; *t*_Run3_(87) = −8.00, *p* < .001; *t*_Run4_(87) = −7.83, *p* < .001 and number group, *t*_Run1_(91) = −16.93, *p* < .001.; *t*_Run2_(90) = −15.82, *p* < .001; *t*_Run3_(91) = −13.66, *p* < .001; *t*_Run4_(91) = −15.90, *p* < .001. This decrease in reaction time for larger numerical distances can be visualized in the average reaction time across the three distances (Figure 6).

**Table 8:** Average accuracy for correct trials for ordinality tasks for the number and letter groups

	Run 1	Run 2	Run 3	Run 4
Letter group	0.96 (0.04)	0.96 (0.04)	0.97 (0.03)	0.96 (0.03)
Number group	0.97 (0.02)	0.97 (0.02)	0.97 (0.03)	0.96 (0.03)

*Note*: Values represent mean accuracy (*SD*).

**Table 9:** Average of the standardized regression coefficients for each group across the four training runs

	Run 1	Run 2	Run 3	Run 4
Letter group	−0.11 (0.09)	−0.09 (0.09)	−0.07 (0.08)	−0.08 (0.09)
Number group	−0.14 (0.08)	−0.14 (0.09)	−0.14 (0.10)	−0.13 (0.08)

*Note*: Values represent mean distance effect (*SD*).

We were also interested in how the letter and number groups compared on the ordinality training tasks (i.e., how the ordinality comparison tasks differed between the groups). Put differently, we wanted to determine whether the number group and letter group differed significantly in their performance on their respective ordinality tasks. More specifically, we used independent *t*‐tests to compare reaction time (for correct trials only) and distance effects between the groups. For this purpose, independent *t*‐tests were used to compare Run 1 between the letter and number groups, as well as Run 4 between the letter and number groups on mean reaction time and distance effects. For mean reaction time, the letter group completed the letter ordinality task significantly more slowly (*M* = 608.08 ms, *SD* = 169.43) than the number group performed the number ordinality task (*M* = 511.07 ms, *SD* = 101.91) for Run 1, *t*(145.34) = 4.67, *p* < .001. Levene's test indicated unequal variances (*F* = 6.32, *p* = .013), therefore degrees of freedom were adjusted from 180 to 145.34. On Run 4, the letter group also performed the letter ordinality task significantly more slowly (*M* = 532.81 ms, *SD* = 93.20) than the number group performed the number ordinality task (*M* = 483.59 ms, *SD* = 100.56), *t*(178) = 3.40, *p* = .001. For the distance effects, the groups demonstrated a significant difference in Run 1, with the letter group showing a significantly smaller distance effect than the number group, *t*(180) = 2.70, *p* = .008. In Run 4, the letter group also showed a significantly smaller distance effect than the number group, equal variances not assumed (*F* = 4.04, *p* = .046), *t*(170.42) = 4.09, *p* < .001. Therefore, the letter ordinality task seemed to be more difficult for participants, as indicated by the higher reaction time.

The letter ordinality task was performed more slowly than the number ordinality. One explanation for this finding could be that in order to complete the letter ordinality task, participants were mapping the letter stimuli onto their respective numerical counterparts (e.g., assigning B to a magnitude of 2), as this would involve an extra step of processing in comparison to the number ordinality task. To ensure that participants were not just using a number magnitude strategy in the letter ordinality task, we compared performance on the letter arithmetic task between the letter and number groups. For the letter arithmetic task, we calculated the average accuracy, reaction time for correct trials and performance for each group (see Table 10). The performance measure was calculated using a formula to combine reaction time and error rate: Performance = Reaction time (1 + 2[Error rate]), where reaction time referred to average response time of both correct and incorrect trials (Goffin & Ansari, 2016; Lyons, Price, Vaessen, Blomert, & Ansari, 2014). We carried out an independent samples *t* test as well as an independent samples Bayesian *t* test for reaction time, accuracy, and performance on the letter arithmetic task. For these analyses, we predicted that the letter and number groups would perform similarly on the letter arithmetic task. Better performance on the letter arithmetic task in the letter training group would imply that participants are using a strategy involving assigning numerical magnitudes to letters (e.g., C = 3) during the ordinality training. This would indicate a use of a numerical cardinality strategy, as opposed to a symbolic ordinality strategy. Similar behavioral performance on the letter arithmetic task in the letter and number groups however, could indicate that the letter group performed the letter ordinality by activating their representations of the ordinal relationships between letters. In other words, we expected to find support for the null hypothesis, and continued data collection until a BF in support of the null indicated strong evidence for no difference between‐groups (BF H_01_ = 6). The use of a BF stopping rule allows the researcher to continue collecting data until a cutoff BF is achieved that signifies the evidence in favor of an alternative or null hypothesis is strong (Marsman & Wagenmakers, 2017). This means that excess data will not be collected, and the strength of the confidence in favor of the hypothesis can be quantified. In the current study, data collection continued until the data were six times more likely under the null hypothesis (no significant difference between the letter and number groups on the letter arithmetic task) than the alternative. This stopping rule was pre‐registered on the Open Science Framework (https://osf.io/s6e7u/). Results from the independent t‐tests indicated that the number and letter groups did not differ significantly in reaction time, accuracy or performance on the letter arithmetic task, *t*(177) = 0.11, *p* = .92, BF_01_ = 6.14; *t*(177) = −0.17, *p* = .86, BF_01_ = 6.09; *t*(177) = 0.31, *p* = .756, BF_01_ = 5.90, respectively. From the results of the Bayesian *t* tests, we can surmise that there is strong evidence for the null hypothesis that the letter and number groups did not differ on the letter arithmetic task (Jeffreys, 1961). More specifically, finding support for the null hypothesis suggests that participants did not assign numerical values to letters in the letter ordinality task, and instead, likely relied on their representations of the ordinal relationships between the letter stimuli to complete the task.

**Table 10:** Mean reaction time (ms), accuracy, and performance on the letter arithmetic task for the letter and number groups

	Reaction time (ms)	Accuracy	Performance
Letter group	6,530.92 (2,262.14)	0.93 (0.07)	7,528.12 (2,868.63)
Number group	6,496.71 (2,034.68)	0.93 (0.07)	7,404.68 (2,431.11)

*Note: SD* is given in brackets.

### 4.3 Discussion

The first goal of Experiment 2 was to determine if the stimuli from the adaptation task in Experiment 1 generated behavioral distance effects when participants were asked to process the ordinal relationships between the symbols. When presented in a passive task, the letter stimuli did not demonstrate a neural parametric effect in Experiment 1. Therefore, it was important that we verified that the letter stimuli used in Experiment 1 generate a behavioral distance effect, and that results from Experiment 1—the lack of a parametric effect for letters—did not occur due to an issue with the stimuli chosen. In Experiment 2, participants in both the number and letter training groups demonstrated distance effects. The symbols chosen were the same as used in the adaptation task in Experiment 1, thereby confirming that at the behavioral level, these letter stimuli generate distance effects. Therefore, even though the letter stimuli did not generate a parametric effect at the whole‐brain level in Experiment 1, the same letter stimuli do generate a distance effect in an explicit task. However, it should be noted that the distance effects obtained from the letter ordinality task were significantly smaller than the number ordinality task, which could indicate that performance on the letter task was not as strongly affected by the ordinal relationships between letters as performance on the number task. Support for the proposal that the ordinal relationships are not as fluent in letters in comparison to numbers also comes from the finding that the letter ordinality task was performed more slowly than the number ordinality task, which fits with previous research (Van Opstal et al., 2008; Vogel, Haigh, et al., 2017).

Although the letter and number groups showed quantitative differences in the magnitude of the distance effects obtained, the finding that both sets of stimuli elicited distance effects in the same pattern—increased response time with decreased distance—provides support for a qualitative similarity between the sets of symbols at the behavioral level.

The second goal of Experiment 2 was to investigate whether the distance effect in the letter ordinality task could have been an artifact of a numerical magnitude assignment strategy. However, there was substantial evidence that the different training groups did not differ on the letter arithmetic task. If the letter group—the group that practiced the letter ordinality task—outperformed the number group, it could be argued that the letter group performed the letter ordinality task using a numeric strategy. More specifically, practice over the four runs of the ordinality task in which they assigned numeric values to letters to complete the task could have led to this group outperforming the number group when asked explicitly to apply a numeric strategy to the letter arithmetic task. However, the two groups scored very similarly on the letter arithmetic task, which suggests that the letter ordinality task was not carried out using a numerical magnitude strategy. Participants seem to instead be performing the letter task by accessing the ordinal relationships between these symbols. However, it should be noted that this interpretation rests on the assumption that there would be transfer in training on the letter ordinality‐training task to the letter arithmetic task. In other words, the assumption is that if the participants were using a numerical assignment strategy in the letter ordinality task, that this would enhance their performance on the subsequent letter arithmetic task. Therefore, there still remains a possibility that participants used a numerical strategy for the letter ordinality task; however, this practice did not result in an advantage on the letter arithmetic task. Further research is needed to disentangle these explanations.

It is unclear what mechanisms underlie the behavioral distance effects observed in both letter and number tasks. Distance effects generated from symbolic numerical tasks are often explained through the ANS theory of number representation; number symbols are mapped onto an analog magnitude system with overlapping representations. However, the theory of the ANS underlying symbolic distance effects is a subject of significant debate. As previously discussed, Van Opstal et al. (2008) demonstrated that a distance effect could be obtained with letter stimuli, a finding that was replicated by the current study. Given that letters are not referents for a quantity system, these behavioral findings of distance effects that are common to both numbers and letters call into question the theory that the ANS theory is necessary or sufficient to explain the distance effects observed with number stimuli.

Alternative mechanisms have been suggested to explain distance effects due to symbolic numerical stimuli. For example, Krajcsi (2017) suggested instead of the ANS, a discrete semantic system (DSS) underlies symbolic number representation. Here, symbolic numbers exist as nodes that are connected through semantic associations. In this account, the NDE is a result of these connections between the number nodes, as opposed to the representational overlap posited by the ANS theory. In support of the DSS view of representation, recent behavioral evidence suggests that the ANS is not sufficient to explain the pattern of responses observed in symbolic numerical comparison tasks (Krajcsi, Lengyel, & Kojouharova, 2018). Instead, the DSS, in which numbers are represented discretely with semantically associated nodes, seems to better fit symbolic numerical comparison behavioral data, and thus may reflect a more suitable explanation for the NDE in symbolic numerical tasks than the ANS. Fitting with this hypothesis that different mechanisms underlie symbolic and nonsymbolic numerical representation, both Krajcsi (2017) and Lyons, Nuerk, and Ansari (2015) did not find a significant association between measures from symbolic and nonsymbolic comparison tasks within‐participants. If these tasks are tapping into representations that have a shared underlying mechanism (i.e., the ANS), one would expect an association between the nonsymbolic and symbolic measures.

In summary, the precise mechanisms underlying distance effects are contested. Although letters and numbers seem to share a similar behavioral signature, in Experiment 1 we found that the response to these same stimuli was quite dissimilar. However, Experiment 2 demonstrated that the lack of a finding of a neural distance effect for letters in Experiment 1 is not because the stimuli list of Experiment 1 cannot generate distance effects, given the finding of a behavioral distance effect for letters in Experiment 2. Instead, it could be hypothesized that different mechanisms underlie behavioral distance effects in forced response tasks, and the neural distance effect in numerical adaptation tasks. Perhaps a response selection mechanism underlies the behavioral distance effects, while a more number‐specific mechanism better fits the neural distance effect (at least in the passive fMR‐A design).

## 5 GENERAL DISCUSSION

Understanding how humans develop the ability to represent magnitude symbolically speaks to more general learning mechanisms that underlie the effects of enculturation. More specifically, having an understanding of how number symbols, as representations that have been constructed over the course of human cultural history, may interact with brain development can give us a greater understanding of how symbol systems in general are accommodated in human neural circuitry.

What mechanisms underlie the distance‐dependent parametric rebound effect that has been reproduced across different studies following adaptation to numerical symbols? What can this effect tell us about symbolic number representation? It is often hypothesized that the symbolic number system is mapped onto an approximate nonsymbolic magnitude system, and that the parametric effect is a signature of this analog system. The current studies tested an alternate hypothesis: whether ordinal relationships between symbols can explain the parametric rebound effect. Contrary to our predictions we found that, in Experiment 1, letters, in contrast to numbers, do not exhibit this neural parametric effect anywhere in the brain during an fMRI adaptation task. However, in Experiment 2, we found that the letters we included in Experiment 1, do elicit a behavioral distance effect. What do these results suggest about symbolic number representation? Several explanations could be offered for the findings from Experiment 1 and 2—behavioral distance effects for both numbers and letters; a neural distance effect only for numbers, including but not limited to:

1. Different mechanisms underlie behavioral distance effects and neural distance effects:
  - Response selection mechanisms lead to behavioral distance effects, and representational overlap leads to the neural parametric effect observed for numbers.
  - Response selection mechanisms lead to behavioral distance effects, and *highly salient* ordinal relationships lead to the neural parametric effect for numbers.
  - Response selection mechanisms lead to behavioral distance effects, and another number‐specific property generates the neural parametric effect for numbers.
2. Different mechanisms underlie number and letter distance effects. A number‐specific mechanism (e.g., representational overlap, salient ordinal relationships, etc.) underlies the number distance effects at both the behavioral and neural level. Differences in the demands on response selection elicit the letter distance effect.
3. Different mechanisms underlie all three effects (i.e., behavioral number distance effects, behavioral letter distance effects, neural number parametric effects).

Further research that empirically investigates the mechanisms underlying neural and behavioral distance effects is necessary to help distinguish between these options. In general, it seems that a level of semantic processing of a symbol is required to generate a neural distance effect; whether or not this is indicative of mapping onto the ANS or some other property of number, remains to be seen. More specifically, the processing of a symbol with an ordered sequence alone is not sufficient to generate a neural parametric effect. This suggests that the system for symbolic number representation may automatically activate more number‐specific properties when presented with a number symbol, as opposed to other more general (in that they also exist for letters) numerical symbol set properties, such as order.

### 5.1 A different response for letters vs. numbers at the neural level

A key question is why did the neural response for numbers and letters differ? Vogel et al. (2019) suggested that the ordinal relationships between numbers may be processed automatically. It could be that ordinal relationships are not as fluent in letters as they are in numbers. In other words, although letters can be arranged as an ordinal sequence (i.e., the alphabet), perhaps this sequence is not activated as automatically as it is for letters. Put differently, when we are presented with a single letter, it could be the case that the letter's place in the ordinal sequence is not activated as automatically as it may be for numbers. Therefore, accessing the ordinal relationships between letters could be a more effortful process that requires an active task. This hypothesis is supported by the finding in Experiment 2 that demonstrates the letter ordinality task was associated with significantly higher reaction times than the number ordinality task. Previous studies have also found longer reaction times in letter processing tasks compared to number processing tasks (Fulbright et al., 2003; Van Opstal et al., 2008; Vogel, Haigh, et al., 2017; Vos, Sasanguie, Gevers, & Reynvoet, 2017).

It is also possible that the parametric distance effect observed in the IPS is not solely related to ordinal relationships between symbols. The present data do suggest that the parametric effect is reflective of some semantic processing of number symbols. However, perhaps symbol‐symbol ordinal relationships are not a good model for the mechanisms underlying the parametric distance effect, and another property of number will provide a better explanation. It may also be possible that the left IPS is more specialized for ordinal relationships in numbers, as opposed to ordinal relationships more generally (e.g., between letters). Further research is needed to address this question.

Another possibility for the lack of parametric effect for letters is that our study was underpowered. However, the number of participants included in the current study was based on previous symbolic numerical adaptation studies that have demonstrated the ratio‐dependent rebound effect. Holloway et al. (2013) included 26 participants (13 participants per group) and found an effect in the left IPS region significant at the whole brain level when using a cluster‐level correction for multiple comparisons set to *p* < .05. Using the same threshold, Vogel, Goffin, et al. (2017) demonstrated parametric left IPS activation using 20 participants. Notebaert et al. (2010) had a sample size of 13. The current study used an adaptation task based closely on these previous studies, and therefore collected a sufficient number of participants to replicate the number parametric effect found in previous research. The fact that we successfully identified parietal regions that demonstrated the expected numerical parametric effect means that our study was sufficiently powered to pick up on this effect, although it is still possible that the effect is present in letters but is much weaker and thus more participants are required to reveal the effect. In support of this prediction, in Experiment 2, we show that letters generate a behavioral distance effect that is significantly smaller than the distance effect for numbers. However, it should also be noted that even at a very liberal, uncorrected threshold, we still did not find a neural parametric effect for letter. Furthermore, Bayesian statistics determined that there was substantial evidence for the absence of the parametric effect for letters within three clusters in the IPS. If the lack of a parametric effect for letters could be attributed to a lack of power to pick up the effect, the Bayesian *t* test would have indicated weak or anecdotal evidence for the null. Although it is difficult to draw conclusions from the absence of an effect, the lack of this effect even at an uncorrected, lenient threshold and the presence of substantial evidence for the null hypothesis supports the notion that there is not a significant neural distance effect for letters in the current study.

Although both numbers and letters have elicited behavioral distance effects, at the neural level the processing of these symbols diverges. In the current study, we did not find a parametric distance effect with letters, but observed this effect for numbers. This finding is somewhat inconsistent with Fulbright et al. (2003). When participants were asked to judge whether letters were in order or not in order, trials that had a smaller numerical distance elicited more activation in several areas including bilateral inferior and middle frontal gyrus and right IPS, compared to trials with a larger numerical distance. The differences between studies in the letter tasks could explain why the current study did not yield distance effect for letters, while Fulbright et al. (2003) did observe some regions demonstrating sensitivity to distance in letters. Fulbright et al. (2003) used an active task requiring participants to select a response, whereas the current study used a passive design. Therefore, differences may arise when participants are asked to explicitly judge the order of a sequence of letters as opposed to viewing letters passively. Since the purpose of our study was to examine symbol representation in the absence of other cognitive processes such as decision making, response selection, and working memory, it is not surprising that our results diverge from an explicit letter‐ordering task. Differences in active versus passive tasks may similarly explain why Attout et al. (2014) found a neural distance effect for a letter‐ordering task in bilateral regions of the IPS.

### 5.2 Hemispheric differences for the number parametric effect

The finding of a left‐lateralized parametric effect in the parietal lobe is consistent with previous number symbol adaptation research (Holloway et al., 2013; Notebaert, Nelis, & Reynvoet, 2010; Vogel et al., 2015; Vogel, Goffin, et al., 2017). In a quantitative meta‐analysis of adaptation studies presenting subjects with symbolic numbers, Sokolowski et al. (2016) found that the left SPL showed a parametric effect for number. In agreement with these results, the current study also found a left‐lateralized parietal cluster for the numerical parametric effect; however, two right‐lateralized parietal clusters were also identified. Right IPS has been found in previous numerical adaptation research (Holloway et al., 2013; Piazza, Izard, Pinel, Le Bihan, & Dehaene, 2004; Vogel et al., 2015). For example, Holloway et al. (2013) found a parametric recovery effect using Chinese numerals in a group of Chinese‐speaking participants. This effect was attributed to a lower familiarity with the Chinese notation when compared to the highly familiar Arabic digit notation (for which this group showed the expected left‐lateralized parametric effect). Vogel et al. (2015) also found parametric modulation of the right IPS with a number symbol adaptation task. A group of children age 6–14 showed a right‐lateralized parametric effect in response to number. The right IPS demonstrated this parametric effect across all ages, while the strength of the left IPS parametric effect was positively correlated with age. As children also have comparatively less experience with number symbols than adults, the involvement of the right IPS may reflect a lower level of fluency with number symbols.

The right IPS may also show parametric modulation when nonsymbolic stimuli are used in an adaptation task or when cross‐format adaptation (number symbols and dot arrays) is used (Piazza et al., 2004, 2007). More specifically, Piazza et al. (2007) presented participants with four conditions (adaptation format‐deviant number format): dots–dots, Arabic–Arabic, dots–Arabic, and Arabic–dots. Brain regions that showed neural recovery that was greater for deviants that were further away from the adapted value compared to closer were identified. Overall, a distance‐dependent recovery effect was observed in parietal regions bilaterally. However, the right parietal cortex showed more distance‐dependent recovery during cross‐notation adaptation. The authors suggested that the right parietal cortex may represent number magnitude symbolically and nonsymbolically in an approximate manner, while the left parietal cortex is refined by number symbol acquisition and offers a more exact representation of magnitudes.

The current study supports the notion of left parietal regions, relative to right parietal areas, as being more strongly involved in fluent, exact symbolic processing, as evidenced by the left parietal clusters identified in the parametric effect contrasts, and specifically in the contrast between the number parametric effect and the letter parametric effect. It is unclear why right IPS clusters were also identified in the contrast parametric_Number_ > baseline, however the finding that the left parietal region seems to be more specified for number processing (the result of the number > letter contrast) is consistent with previous research. The contributions of the left vs. right IPS to symbolic numerical processing is still a topic of investigation in the literature.

### 5.3 Conclusions

To date, it has been unclear whether the correlation between symbolic number processing and the IPS reflects the processing of numerical magnitude, ordinal information or a combination of the two. The findings reported above do not provide evidence in support of the notion that the representation of general (across stimulus categories) ordinal relationships explains the neural parametric distance effect observed for numerical symbols. Consistent with previous literature, several parietal clusters were found to be modulated by numerical distance when participants were shown symbolic numbers. Specifically, the left IPL seems to show specificity for the number parametric effect. However, no regions exhibited such a parametric distance effect for letters. These results therefore do not provide support for the alternative to the most common hypothesis that symbolic number is mapped onto a noisy nonsymbolic magnitude system, which generates the parametric distance effects. However, it could be the case that symbol–symbol relationships are not as fluent in letters as they are in numbers and therefore are not activated during passive adaptation to letters. Further research is needed to investigate the nature of neural number representation.

## Data Availability

The data that support the findings of Experiment 1 of this study are available on request from the corresponding author. The data are not publicly available due to privacy or ethical restrictions. The data that support the findings of Experiment 2 of this study are openly available in the Open Science Framework (Goffin, Slipenkyj & Ansari, 2019) at [https://osf.io/s6e7u/].
